# Force of tuberculosis infection among adolescents in a high HIV and TB prevalence community: a cross-sectional observation study

**DOI:** 10.1186/1471-2334-11-156

**Published:** 2011-06-01

**Authors:** Keren Middelkoop, Linda-Gail Bekker, Hua Liang, Lisa DH Aquino, Elaine Sebastian, Landon Myer, Robin Wood

**Affiliations:** 1 Desmond Tutu HIV Centre, Institute of Infectious Diseases and Molecular Medicine, University of Cape Town, Cape Town, South Africa; 2 Department of Medicine, University of Cape Town, Cape Town, South Africa; 3 Department of Biostatics and Computational Biology, University of Rochester Medical Center, Rochester, USA; 4 Centre for Infectious Diseases Epidemiology & Research, School of Public Health & Family Medicine, University of Cape Town, Cape Town, South Africa; 5 International Centre for AIDS Care and Treatment Programs and Department of Epidemiology, Mailman School of Public Health, Columbia University, New York, USA

## Abstract

**Background:**

Understanding of the transmission dynamics of tuberculosis (TB) in high TB and HIV prevalent settings is required in order to develop effective intervention strategies for TB control. However, there are little data assessing incidence of TB infection in adolescents in these settings.

**Methods:**

We performed a tuberculin skin test (TST) and HIV survey among secondary school learners in a high HIV and TB prevalence community. TST responses to purified protein derivative RT23 were read after 3 days. HIV-infection was assessed using Orasure® collection device and ELISA testing. The results of the HIV-uninfected participants were combined with those from previous surveys among primary school learners in the same community, and force of TB infection was calculated by age.

**Results:**

The age of 820 secondary school participants ranged from 13 to 22 years. 159 participants had participated in the primary school surveys. At a 10 mm cut-off, prevalence of TB infection among HIV-uninfected and first time participants, was 54% (n = 334/620). HIV prevalence was 5% (n = 40/816). HIV infection was not significantly associated with TST positivity (p = 0.07). In the combined survey dataset, TB prevalence was 45% (n = 645/1451), and was associated with increasing age and male gender. Force of infection increased with age, from 3% to 7.3% in adolescents ≥20 years of age.

**Conclusions:**

We show a high force of infection among adolescents, positively associated with increasing age. We postulate this is due to increased social contact with infectious TB cases. Control of the TB epidemic in this setting will require reducing the force of infection.

## Background

Tuberculosis (TB) remains a major cause of morbidity and mortality in the world[1]. In order to develop effective intervention strategies for TB control, it is important to understand TB transmission in high burden settings. While there have been recent studies assessing TB infection in young children[2-5], there are few data assessing TB infection in older children and adolescents in communities with high TB and HIV burdens[6].

Incidence of TB infection is a measure of current transmission in a community. While repeated testing of uninfected individuals over time is a conventional method for determining incidence of a disease, this methodology is both labour and time intensive, and is further complicated by the boosting of the immune response in immunology-based tests such as the tuberculin skin test (TST)[7,8]. Therefore alternative approaches for calculating incidence from prevalence data have been developed. The annual risk of TB infection (ARTI), calculated from TB infection prevalence data, is an averaged measure of risk of TB infection over the lifetime of the study participants[7,9]. The limitation of this measure is that ARTI only provides an estimate of current transmission or incidence if calculated in very young participants. In comparison, force of infection, defined as the proportion of susceptible individuals that have become infected with *Mycobacterium tuberculosis *in a specified period, can be calculated using changes in age-specific prevalence rates[10-12], and provides an estimate of recent infection incidence across a wider age range.

This approach is a relatively new concept that has been utilized to estimate incidence in diseases in which true incidence is difficult or costly to measure, such as glaucoma[13] and more recently, HIV[12,14]. The mathematical techniques utilized are based on the principle that prevalence is a function of incidence and duration of illness or infectivity[15]. This principle holds true under the assumptions of disease stability. Data from this community suggest that TB transmission has remained relatively constant over the past decade, as evidenced by the stable TB notification rates among HIV-uninfected adults from 1997 to 2008[16], as well as the stable childhood TB over the same time period[2].

We performed a TST survey among adolescents in a high HIV and TB prevalence community to assess prevalence of TB infection and force of infection by age.

## Methods

This study was the third in a series of cross-sectional tuberculin skin testing surveys performed among school-attending children in the study community. The first two surveys were performed in the local primary school in 2006 and 2007[2]. The survey reported in this manuscript was performed in children attending secondary school in the study community in 2009. The same methodology was used in all three surveys, with the addition of HIV testing in the secondary school survey. Children were eligible if they were resident in the community and registered at the local secondary school. Parental consent, and assent from participants <18 years of age, were obtained prior to enrolment. Adolescents ≥18 years of age provided written consent. Basic demographic information was collected and participants were examined for the presence of a BCG scar. Participants received the TST regardless of BCG scar status.

The WHO-recommended standard Mantoux test of 2TU of PPD (Purified Protein Derivative) RT23 with Tween 80 (Statens Seruminstitut, Copenhagen) was administered intradermally to the volar surface of the left forearm by a trained nurse. The tuberculin reaction size was read by a trained assessor three days following the inoculation. The presence or absence of a reaction was noted, and, where present, the size of the induration was measured along perpendicular axes using standard calipers. Participants also provided an oral transudate specimen for HIV testing, using the Orasure^® ^collection device and Vironostika Uni-Form II HIV-1 and HIV-2 plus 0 ELISA test (bioMérieux SA, Marcy l'Etoile, France). HIV results were anonymous but linked to TST results and all adolescents were encouraged to have separate voluntary counseling and testing at local facilities. HIV testing was not performed in the primary school surveys[2].

This study was approved by the University of Cape Town's Research Ethics Committee. All children with a TST reaction ≥10 mm were recalled for investigation for active tuberculosis and referred to the local clinic for further management if appropriate.

Data were analyzed using STATA 9.0 (StataCorp, College Station, Texas). Analysis was performed in two parts: firstly on the secondary school dataset, excluding HIV-infected individuals, and secondly on the secondary school dataset combined with the primary school dataset, excluding the HIV-infected individuals from the secondary school survey. Children who declined HIV testing were considered to be HIV-uninfected for the purposes of the TB analysis.

TST results were calculated as the mean of the two diameters of the TST reaction: a positive reaction was defined at 10 mm cut-off, based on clinical guidelines[17,18]. ARTI was calculated as 1-(1-prevalence)^1/(mean age+0.5)^[19]. As the age in full years at participants' last birthday was used, 0.5 was added to the mean age for the calculation of ARTI[19]. The secondary school sample was divided into age quartiles and TB prevalence and ARTI were calculated overall and for each age group. Bivariate analyses employed Student's t-, Fisher's exact and Wilcoxon sum rank tests, as appropriate. Multiple logistic regression models were developed to examine factors associated with positive TST results.

### HIV data secondary school participants

Univariate and multivariate logistic regression models were developed to determine the demographic characteristics associated with HIV, and to assess for an association between HIV and TB infection. In these models, a TST reaction size of ≥5 mm was used as a positive cut-off for TB infection in HIV-infected adolescents, in keeping with clinical guidelines[17,18].

### Subset of repeat TST participants

Overall, 159 students who had participated in the 2007 survey also took part in the secondary school survey, resulting in repeat tuberculin testing of these participants. Repeated TST tests may be associated with boosting of the immune response, complicating the interpretation of the second TST reaction size[7,8]. We, therefore, analyzed this subset of participants separately from the rest of the cohort. In keeping with existing literature, we defined a true conversion to TST positivity between the two surveys as a change from a negative result (<10 mm) on original TST to a positive result (≥10 mm) on the second test, with an absolute reaction size increase of at least 6 mm[20-22]. Bivariate analyses used Wilcoxon sum rank and Student's t tests, as appropriate for comparison of participants with repeated TST compared to first time participants in 2009. A multivariate regression model was developed to compare reaction sizes between the two groups. The McNemar test was used for matched comparison of 2007 and 2009 TST results.

### Combined surveys database

In order to assess the effect of age on TB prevalence and force of infection, we combined the primary and secondary survey datasets, excluding HIV-infected participants from 2009, as well as the second test in those participants who had repeat tests in 2009. The participants were divided into age quartiles and TB prevalence and ARTI were calculated overall and for each age group.

Smoothed prevalence of TB infection by age was calculated from predictive logistic regression models on the combined primary and secondary school dataset, overall and stratified by gender, excluding those who tested HIV-infected in the secondary school. Force of infection was calculated at specific ages for the pool of individuals who remained uninfected [annual change in prevalence/(1-prevalence)]. Trends in ARTI and force of infection were assessed using Cox-Stuart test for trend[23,24].

For all analysis, 95% confidence intervals (CI) were based on the Poisson distribution and all statistical tests were 2-sided at alpha = 0.05.

## Results

**Figure 1:**
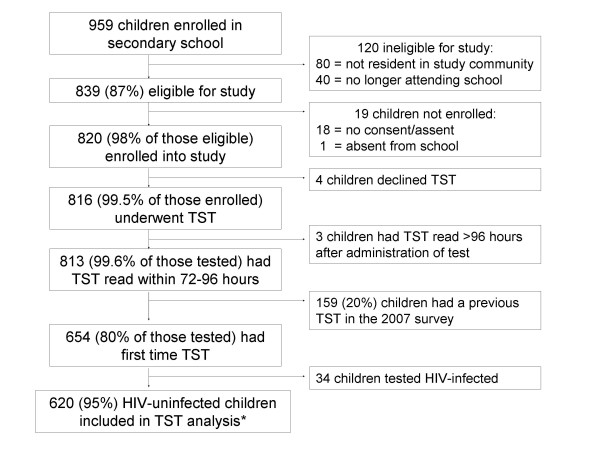
**Consort Diagram for Secondary School Cohort**. * including the 3 participants who declined HIV testing

Of the 959 children enrolled in the secondary school, 839 were eligible for study participation (87%). Ineligibility was due to residence outside of the community (n = 80) or having dropped out of school (n = 40). Consent/assent was obtained for 820 children (98% of those eligible). Refusal by parent or learner accounted for 18 non-consenters and 1 child was not consented due to absenteeism over the study period. All 820 consented students were enrolled. Four of the children enrolled declined tuberculin skin testing, and four children declined HIV testing. Of the 816 children who underwent skin testing, 813 (99.6%) had the TST reaction read within 72-96 hours (Figure 1). No study-related adverse events were noted in the three children assessed outside the window period and these participants were excluded from the analysis.

Table 1 shows the demographic characteristics of the secondary school study cohort. Of the 813 participants who completed TST testing, 159 had received tuberculin skin testing in the 2007 survey in the community. These participants were excluded from the main analysis and their results are presented in a sub-analysis. Of the remaining 654 children, all but three of the participants underwent HIV testing. In total 34 participants of the remaining participants tested HIV-infected, and were excluded from the TB analysis (n = 620; Figure 1).

**Table 1:** Demographic characteristics of the secondary school sample.

	**First time TST participants**^Φ^			**Repeat TST participants**^ΦΦ^		
	HIV-uninfected	HIV-infected	Total	HIV-uninfected	HIV-infected	Total
	n = 617	n = 34	n = 654	n = 154	n = 4	n = 159
	*n (%)*	*n (%)*	*n (%)*	*n (%)*	*n (%)*	*n (%)*
**Age: mean (range)**	17.5 (13-22)	18.4 (15-22)	**17.5 (13-22)**	15.4 (13-19)	15.3 (14-17)	**15.4 (13-19)**
**13-16 yrs**	176 (29)	5 (15)	**183 (28)**	133 (86)	3 (75)	**136 (86)**
**17 yrs**	148 (24)	4 (12)	**152 (23)**	13 (8)	1 (25)	**15 (9)**
**18 yrs**	112 (18)	9 (26)	**121 (19)**	6 (4)	0 (0)	**6 (4)**
**19-22 yrs**	181 (29)	16 (47)	**202 (30)**	2 (1)	0 (0)	**2 (1)**
**Gender: Male**	255 (41)	9 (34)	**266 (41)**	81 (53)	2 (50)	**84 (53)**
**BCG Scar present**	80 (13)	6 (18)	86* **(13)**	32 (21)	0 (0)	**32 (20)**
**TST positive**	331 (54)	11 (32)	**345 (53)**	90 (58)	1 (25)	**91 (57)**

*n = 653: one participant's BCG scar status was not recorded

^Φ ^Three participants declined HIV testing

^Φ Φ ^One participant declined HIV testing

Among the 620 participants, ages ranged from 13 to 22 years, with a mean age of 17.5 years, and 59% of participants were female. The majority of the children did not have a BCG scar (87%); one child's BCG scar status was not recorded, and this participant was excluded from analysis involving BCG scar status.

**Figure 2:**
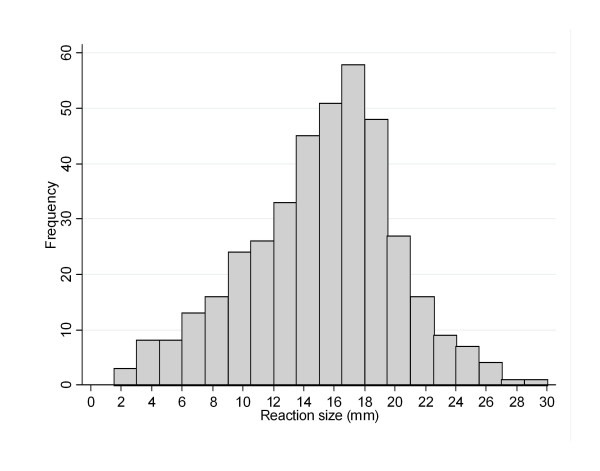
**Frequency distribution of TST reactions (>0 mm) in HIV-uninfected participants in secondary school survey**.

TST reaction sizes ranged from 0 to 30 mm (median = 11.5 mm; IQR: 0-16.5 mm), and 222 children had a TST result of 0 mm (36%). The frequency distribution of reaction sizes >0 mm are presented in Figure 2. There was no significant difference between the median size of reactions in participants with BCG scars compared to those without scars (11 vs 11.5 mm respectively, p = 0.24).

At the 10 mm cut-off 334 participants (54%) had a positive TST result. There was no significant difference in the defined TB positivity by BCG scar status (p = 0.99). In a multivariate logistic regression model predicting the relative odds of a positive TST result, age was positively associated with a positive TST result (adjusted odds ratio (OR) for a 1-year increase in age: 1.10, 95% CI: 1.01 - 1.21; p = 0.03), as was male gender (adjusted OR for female compared to male: 0.65, 95% CI: 0.47-0.90; p = 0.01).

The overall ARTI for this sample was 4.2% (95% CI: 3.8-4.7%). Table 2 reports TB prevalence and ARTI overall, and by age quartiles. The ARTI did not differ significantly across the age quartiles (p = 0.75) or across age by years from 14 to 21 years of age (p = 0.63).

**Table 2:** Secondary school TB prevalence and ARTI by age quartile for the 10 mm cut-off point (excluding HIV-infected and repeat TST participants):

Age Category	Mean Age*	n (%)	TST positive	Prevalence	ARTI% (95% CI)
13-16 yrs	15.8	178 (29)	85	47.8%	4.0% (3.2-5.0%)

17 yrs	17.5	148 (24)	76	51.4%	4.0% (3.2-5.0%)

18 yrs	18.5	112 (18)	58	51.8%	3.9% (2.9-5.0%)

19-22 yrs	20.2	182 (29)	115	63.2%	4.8% (4.0-5.8%)

**TOTAL**	**18**	**620**	**334**	**53.9%**	**4.2% (3.8-4.7%)**

* The mean age reported here is the age used for the ARTI calculations, and is based on the mean age for the age category+0.5 years, as described in the methods section.

Note: Participants who declined HIV testing were presumed to be HIV-uninfected and were included in the TB analysis

### Sub-analysis: HIV and TB infection

Of the 820 participants enrolled in the secondary school survey, 816 (99.5%) consented to HIV testing. In total 40 (4.9%) participants tested HIV-positive. In a logistic regression model, HIV infection was associated with increasing age (OR: 1.3; 95% CI: 1.1-1.6; p = 0.001) and female gender (OR: 2.3; 95% CI: 1.1-4.7; p = 0.03).

Among the 809 participants who underwent both TST and HIV testing, 38 were HIV-infected, four of whom had previous TST testing in 2007. The median TST reaction size among HIV-infected participants (amongst those who did not have repeat TST assessment; n = 34) was smaller than that of HIV-uninfected participants (0 vs 11.5 mm; p = 0.08), and this difference was significant when adjusted for age and gender (p = 0.04). Using the revised cut-off of ≥5 mm, 15 (40%) HIV-infected participants were TST positive. In a multivariate logistic regression model adjusted for age and gender, HIV infection was not significantly associated with TST positivity (OR = 0.53; 95% CI: 0.27-1.05; p = 0.07).

### Sub-analysis: Repeated TST testing

In 2009, 159 students who had participated in the 2007 survey took part in the secondary school survey, four of whom were HIV-infected. The mean reaction size in 2009 among participants with repeat tests was larger compared to participants who tested for the first time (15 vs 11.5 mm; p < 0.001), and this finding persisted when adjusted for age and gender (p < 0.001). Overall, TB prevalence did not differ between those that had a repeat TST test and those participants who were testing for the first time (58 vs 54%, p = 0.29).

Using a 10 mm cut-off for positivity, in 2007, 76 (48%) of the 159 participants had a positive TST result; in 2009 91 (57%) tested TST positive. Overall, 19 of the 83 participants who were TST negative in 2007 tested positive in 2009 (p = 0.003), and four (5%) reverted to a TST negative result. Using the definition outlined above, 18 of the 19 convertors had an increase in reaction size of ≥6 mm and were therefore considered true new infections with TB. These 18 participants, from the pool of 83 susceptible children in 2007, equate to an incidence of 22% over two years (95 CI: 13-32%), or an annual incidence of infection of 11% ([18/83]/2).

### Combined Primary and Secondary school database

In order to assess the effect of age on TB prevalence and force of infection, we investigated combining the primary and secondary school surveys. We compared the prevalence of TST positive results for the three cohorts in the overlapping age ranges of 10 to 12 years and 14 to 16 years. The chi-squared test for comparison was not significant (survey 1 and 2: p = 0.66; survey 2 and 3: p = 0.46), nor was survey year a significant risk factor in multivariate regression model for TST positivity (p = 0.23). Therefore we combined these two datasets. In the primary school survey, TST readings were available for 831 of the 832 children enrolled. Therefore the combined database, excluding the participants who tested HIV-infected, was comprised of 1,451 participants.

Ages of the combined cohort ranged from 5 to 22 years, with a mean age of 13.6 years (standard deviation = 4.1), and 52% of participants were female. The majority of the children did not have a BCG scar (80%); BCG scar status was not available for two participants and they were therefore excluded from analysis involving BCG scar status.

**Figure 3:**
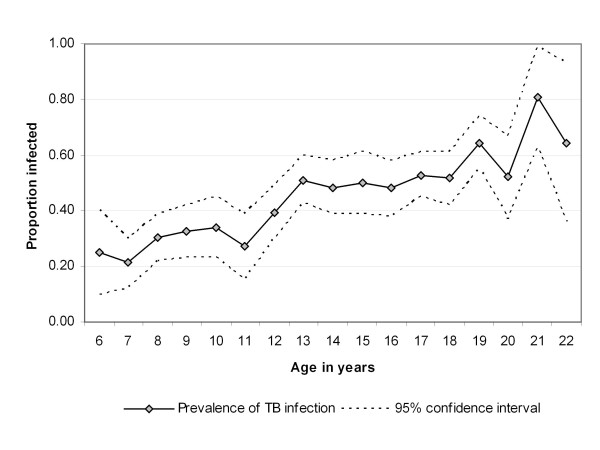
**TB prevalence in combined dataset, by age (in years)**. Note: This figure is based on the unadjusted data presented in Table 3.

TST reaction sizes ranged from 0 to 30 mm (mean = 8.0; median = 0 mm). Overall 728 (50%) of the participants had no reaction to the TST. At the 10 mm cut-off 645 participants (45%) had a positive TST result (Table 3). TB prevalence by age is presented in Figure 3. In a multivariate logistic regression model a positive TST result was significantly associated with age (adjusted OR for a 1-year increase in age: 1.17, 95% CI: 1.10 - 1.25; p < 0.001) and male gender (adjusted OR for female compared to male: 0.74, 95% CI: 0.60-0.92; p = 0.01). TST positivity was not associated with BCG scar status (p = 0.40).

**Table 3:** TB prevalence and ARTI by age quartile for the 10 mm cut-off point for the three surveys combined (excluding HIV-infected and second tests in repeat TST participants):

Age Category	Mean Age*	n (%)	TST positive	Prevalence	ARTI% (95% CI)
**Age Category**					

5-9 yrs	8.3	325 (22)	91	28.0%	3.9% (3.1-4.7%)

10-14 yrs	12.8	488 (34)	207	42.4%	4.0% (3.2-5.0%)

15-17 yrs	16.7	344 (24)	174	50.6%	3.9% (2.9-5.0%)

18-22 yrs	19.6	294 (20)	173	58.8%	4.8% (4.0-5.8%)

**Gender**					

Male	13.8	691 (48)	330	47.8%	4.6% (4.1-5.1%)

Female	14.3	760 (52)	315	41.4%	3.7% (3.3-4.1%)

**TOTAL**	**14.1**	**1,451**	**645**	**44.5%**	**4.1% (3.8-4.4%)**

* The mean age reported here is the age used for the ARTI calculations, and is based on the mean age for the age category+0.5 years, as described in the methods section.

Note: Participants who declined HIV testing were presumed to be HIV-uninfected and were included in the TB analysis

Table 4 reports the smoothed prevalence of TB infection by age, as predicted by our logistic regression model. Also reported in Table 4 are the ARTI and force of infection by age, calculated from the smoothed prevalence. The overall ARTI for this sample was 4.1% (95% CI: 3.8-4.4%). The ARTI did not differ significantly across age by years from 6 to 22 years of age (p = 0.15). The force of infection increased with increasing age, and this trend was significant (p = 0.01).

**Table 4:** Predicted prevalence, ARTI and force of infection by age in study cohort

Age	Number of participants	Predicted Prevalence* (95% CI)	ARTI**	**Force of infection **^Φ^
5	1	0.22 (0.17 - 0.27)	4.85	

6	36	0.24 (0.19 - 0.29)	4.47	2.93

7	84	0.26 (0.22 - 0.30)	4.21	3.15

8	112	0.28 (0.24 - 0.32)	4.06	3.39

9	92	0.31 (0.27 - 0.34)	3.96	3.81

10	71	0.33 (0.29 - 0.36)	3.91	3.96

11	59	0.35 (0.32 - 0.39)	3.89	4.28

12	107	0.38 (0.35 - 0.41)	3.91	4.47

13	135	0.41 (0.38 - 0.43)	3.93	5.03

14	116	0.43 (0.41 - 0.46)	3.97	5.11

15	84	0.46 (0.44 - 0.49)	4.04	5.58

16	106	0.49 (0.46 - 0.52)	4.10	5.70

17	154	0.52 (0.48 - 0.55)	4.18	6.05

18	112	0.54 (0.51 - 0.58)	4.27	6.44

19	101	0.57 (0.53 - 0.61)	4.36	6.63

20	46	0.60 (0.55 - 0.64)	4.45	7.11

21	21	0.62 (0.58 - 0.67)	4.55	7.37

22	14	0.65 (0.60 - 0.70)	4.66	7.34

* Predictive logistic regression model: logit{P(Age)} = -1.82+0.111 age.

** The ARTI calculations are based on the mean age for the age category+0.5 years, as described in the methods section.

^Φ ^Force of infection = annual change in prevalence/(1-prevalence)

## Discussion

This is one of the first studies to report TB infection prevalence, ARTI and force of infection in adolescents in a high TB and HIV prevalent setting.

In this study we confirmed the high ARTI rate reported in the primary school children[2], and show a high force of infection. ARTI provides a measure of the averaged risk of infection over the participants' lifetime. Therefore this measure pertains to the annual risk over a period of up to 20 years, and consequently the ARTI provides little information on the current transmission within the study population.

Force of infection is a measure of recent transmission and the high rates reported here are in keeping with the substantial burden of TB prevalence[25] and notifications[26] in this community and in South Africa[1]. We demonstrated that force of infection was positively associated with increasing age. These findings are consistent with those reported prior to the HIV epidemic[27], as well as by more recent mathematical modeling[6]. The advantage that this study had over the modeling paper is that of greater numbers of older adolescent participants all recruited from the same community, and the ability to show that the force of infection continues to increase up to approximately 20 years of age. In adulthood force of infection becomes harder to measure due to a reduced proportion of susceptible individuals, and the inability to identify secondary infections.

Force of infection is a function of the probability of an effective encounter with an infectious TB case, and as such is a product of TB disease prevalence and mixing patterns. We postulate that the association between force of infection and age may be due to increasing social mixing patterns, resulting from changing social interactions associated with age. We have previously shown that TB infection in primary school children is associated with an adult TB case on their residential plot[28], while TB transmission between adults is due to social interactions off the residential plots[6]. Data from Europe shows that number of social contacts peak in adolescents[29], suggesting that the likelihood of contact with infectious persons may also peak at this time. The social interactions, and therefore risk of TB infection, of mid-teens may more closely resemble that of adults rather than younger children in the community.

We have also shown a changing risk of TB infection by gender: in the primary school surveys, gender was not associated with TB infection[2], but in the secondary school survey, male gender was associated with increased TB infection. As males get older, their risk of TB infection out-strips that of females of similar age, and this is consistent with other reports in the literature[9,21]. This increased risk may be due to a combination of increased biological susceptibility, differing immunological responses or differences in socialization patterns of male compared to female adolescents.

The lack of association between HIV and TB infection is a key finding: adolescents infected with HIV did not appear to be at higher risk for acquiring primary TB infection. This is in keeping with findings reported from lower HIV and TB prevalent settings[30,31], and consistent with studies that suggest the establishment of TB infection is mechanistic, as opposed to immune-based[32]. However, adolescents are at increased risk of progression to TB disease in the first two years following TB infection[33], and the high infection rates in this community place adolescents at substantial risk of TB disease.

The high incidence rate of TB infection in the subset of participants tested 2 years apart, confirms the high force of infection. However, due to the boosting of the immune response with enhanced allergy noted with repeated TSTs[8,34-38], the incident rate in this subset is higher than the force of infection. This is highlighted by the larger median TST reaction sizes in participants with a repeat test compared to first time participants. Similarly, reduced and anergic responses to TST have been noted in HIV-infected patients[39-42], as evidenced by the smaller median reaction sizes in HIV-infected participants compared to HIV-uninfected participants. These two scenarios highlight a limitation of using an immune-based test for determining TB infection.

Only 20% of the children in this study had an observable BCG scar, despite the South African policy to vaccinate all infants with the BCG vaccine[43]. However BCG scarring may be variable[38,44] and we found no difference in the distribution of TST results between those children with or without BCG scars.

In this study, only 1% of TST results were weakly positive (1 to 5 mm), suggesting minimal cross reaction with environmental mycobacteria[19]. Given the high TB prevalence in the sample, the positive predictive value of the TST is likely to be high.

HIV testing was not performed in the primary school surveys. However, the Actuarial Society of South Africa (ASSA) 2003 AIDS and Demographic model for the African population[45,46] reports an HIV prevalence of <4% in this age group. Therefore HIV is unlikely to have substantially impacted TST readings in this group and, given the anergic reactions to TST associated with HIV[39-42], any impact of HIV is likely to result in an underestimate of TB infection. We were able to exclude HIV-infected children from the secondary school TB analysis, thereby reducing the potential bias resulting from dual infections.

This study had a very low refusal rate (<4% across all three surveys), and as such volunteerism is unlikely to have biased our results. However, it should be noted that the school-attending children may not be representative of all the children in the community, in particular with regards to risk of HIV infection[47,48]. However, should either HIV or TB infection be higher among non-school attending children, this would result in an underestimation of the prevalence and force of TB infection in this study.

The high force of infection in this community would result in significant rates of primary and secondary TB infection and, given the high HIV prevalence among adults[6], and the considerable increased risk of progression to TB disease in HIV-infected individuals[30,49], these findings could explain the substantial incidence of TB disease in this setting. Control of the TB epidemic requires an increasing proportion of non-infected individuals in a population, in other words, a decreased force of infection[50]. In order to reduce the force of infection, National Tuberculosis Programmes need to decrease the prevalence of infectious cases in the community. We have previously shown that a high coverage antiretroviral treatment (ART) program will reduce TB prevalence among HIV-infected participants, due to both improved immune function and the active TB case-finding among patients initiating ART[25]. Extending active case finding to HIV-uninfected residents may substantially reduce the burden of infectious cases. Social programs, such as improved housing, that impact the environment in which individuals interact may also lead to a reduction in the incidence of TB infection.

## Conclusions

In conclusion, these data suggest a substantial force of infection among adolescents, which is associated with increasing age. This is most likely due to changing social mixing patterns among adolescents, resulting in increased contact with infectious TB cases. Studies into social interaction patterns at different ages in this setting may help to better understand this increasing risk of TB infection. HIV infection was not associated with increased risk of TB infection. However, the extremely high force of infection, together with the high community HIV prevalence, explains the overwhelming burden of TB disease in this township. Control of the TB epidemic will require reducing the force of infection, and further studies assessing intervention strategies such as those suggested here are required.

## Competing interests

The authors declare that they have no competing interests.

## Authors' contributions

KM, LGB, RW, LM and LDHA designed the study. KM, LDHA and ES collected the data. KM did the analyses with input from RW, LM and HL. KM wrote the paper with input from all the authors who each approved the final version.

## Pre-publication history

The pre-publication history for this paper can be accessed here:

http://www.biomedcentral.com/1471-2334/11/156/prepub
